# Psychosocial working conditions and mental health among medical assistants in Germany: a scoping review

**DOI:** 10.1186/s12889-024-17798-2

**Published:** 2024-03-06

**Authors:** Kira Schmidt-Stiedenroth, Viola Mambrey, Annegret Dreher, Adrian Loerbroks

**Affiliations:** Institute of Occupational, Social and Environmental Medicine, Centre for Health and Society, Medical Faculty and University Hospital, Heinrich Heine University Düsseldorf, Moorenstr. 5, 40225 Düsseldorf, Germany

**Keywords:** Medical assistants, Psychosocial working conditions, Mental health, Germany

## Abstract

**Background:**

Medical assistants (MA) constitute one of the largest professions in outpatient health care in Germany. The psychosocial working conditions of health care staff are generally believed to be challenging and to thereby increase the risk of poor mental health. A review of MA’s psychosocial working conditions and mental health is lacking, however. We aimed to systematically identify and summarize existing research on psychosocial working conditions and mental health of MA by addressing (1) Which methods, concepts, and instruments have been used to capture the psychosocial working conditions and mental health among MA in Germany? (2) What findings are available? and (3) What are the research gaps?

**Methods:**

We systematically searched Medline, Scopus, CCMed and Google Scholar. Using the Population Concept Context (PCC)-framework, we applied the following eligibility criteria: (a) Language: English or German, (b) publication between 2002-2022, (c) original study, (d) study population: mainly MA (i.e., ≥ 50% of the study population), (e) concept: psychosocial working conditions and/or mental health, and (f) context: Germany. Two reviewers extracted data independently, results were compared for accuracy and completeness.

**Results:**

Eight hundred twenty-seven sources were identified. We included 30 publications (19 quantitative, 10 qualitative, and one mixed methods study). Quantitative studies consistently reported high job satisfaction among MA. Quantitative and mixed methods studies frequently reported aspects related to job control as favorable working conditions, and aspects related to job rewards as moderate to unfavorable. Qualitative studies reported low job control in specific work areas, high demands in terms of workload, time pressure and job intensity, and a desire for greater recognition. Social interactions seemed to be important resources for MA. Few studies (*n* = 8) captured mental health, these reported inconspicuous mean values but high prevalences of anxiety, burnout, depression, and stress among MA. Studies suggested poorer psychosocial working conditions and mental health among MA during the COVID-19 pandemic.

**Conclusions:**

Quantitative studies tend to suggest more favorable psychosocial working conditions among MA than qualitative studies. We suggest mixed methods to reconcile this alleged inconsistency. Future research should examine discrepancies between job satisfaction and unfavorable working conditions and if psychosocial working conditions and mental health remain changed after the COVID-19 pandemic.

**Supplementary Information:**

The online version contains supplementary material available at 10.1186/s12889-024-17798-2.

## Background

In Germany’s decentralized primary health care system, medical assistants (MA) are the first point of contact for patients in primary care practices [1]. MA constitute one of the largest occupational groups in outpatient care in Germany [2]. With less than 4% men employed as MA in Germany, the profession is mainly female [2]. Although a popular choice for vocational training, the drop-out rates are very high both before and after completing the MA qualification [3]. For instance, a study reported that roughly a quarter of the non-physician healthcare workers who completed vocational training in 2019 and 2020 left the primary care practices even though they were offered a contract [4]. Among those, 18,7% declared to have switched to inpatient settings and 19,6% to a different profession [4]. Another study reported that around a third of surveyed primary care practices claimed to have been affected by the drop-out of MA from vocational training between 2017 and 2021 [5]. The primary care practices reported mainly personal reasons, followed by a lack of social skills and not fitting to the team as explanations for the drop-out from vocational training [5].

The range of MA’s professional activities include assisting physicians in diagnostic procedures and treatments (e.g. x-ray imaging, wound care, blood collection), carrying out administrative tasks such as the documentation and management of patients’ files, billing, scheduling appointments, organizing work processes in the practice, and keeping an overview of the available consumables [6]. The versatility of the job profile requires various team-related, organizational, and medical skills while demanding a high degree of responsibility and diligence towards the health care and safety of patients [7]. The MA profession can further be regarded as a customer/patient oriented profession involving interactional and emotional work demands [8–10].

Available evidence indicates that unfavorable psychosocial working conditions are, in general, associated with poorer (mental) health outcomes [11, 12]. In the case of the health care workforce, psychosocial working conditions and experienced stress have been further linked with diminished quality of care and poorer outcomes in patients’ health [13–16]. Specifically among MA, work stress has been associated with frequent self-reported slips and lapses and poorer interaction with patients [17]. Additionally, it has been suggested that unfavorable psychosocial working conditions such as leadership, job strain and job control, as well as poor mental health, can predict intended and actual turnover in health care professions [18, 19].

The consequences of particular psychosocial job characteristics, i.e. their possible impact on mental health, patient care as well as intended or actual turnover are relevant for the MA profession in the context of the German health care system, especially in view of the current shortage of skilled workers [20]. Thus, the examination of psychosocial working conditions and mental health among MA constitutes a research endeavor of utmost importance. In contrast to other large occupational groups in health care (e.g., nurses and physicians), the psychosocial working conditions and mental health among MA in Germany have only become the focus of research in recent years. A comprehensive review of this research is lacking to date, however.

### Objectives

We aimed to systematically identify and summarize existing original data on the psychosocial working conditions and mental health among MA in Germany. We have focused on Germany due to the regional specificity of the profession. To achieve our objective, we addressed the following research questions: 1) Which methods, concepts, and instruments have been used to capture the psychosocial working conditions and mental health among MA in Germany? 2) What findings are available with regard to the psychosocial working conditions and mental health among MA in Germany? and 3) What are the research gaps pertaining to the psychosocial working conditions and mental health among MA in Germany? We applied the PCC-Framework (Population: MA, Concept of interest: psychosocial working conditions or/and mental health among MA, Context: Germany) recommended for scoping reviews [21] to conceptualize our research questions. We decided to conduct a scoping review, as it is a better suited method to identify available evidence and knowledge gaps when the body of literature is heterogeneous and thus not apt for a more precise, comprehensive systematic review [21, 22].

## Methods

As recommended [21], we developed a scoping review protocol a priori and registered it with the Open Science Framework (https://osf.io/8sev4). Following the protocol, the review was carried out based on the following steps: (1) the identification of research questions, a framework to address them, and the selection of criteria for inclusion and exclusion, (2) the identification of relevant databases and additional sources, (3) the development of the search strategies, (4) the systematic search of the selected databases and additional sources, (5) the selection of eligible sources, (6) data extraction (charting), and (7) data synthesis and summary. The review was conducted by a multidisciplinary team including members with broad experience in epidemiological research on working conditions among MA (VM, AD, AL) using the Preferred Reporting Items for Systematic Reviews and Analyses Extension for Scoping Reviews (PRISMA-ScR) guidelines [23].

### Eligibility criteria

We sought to include all types of original studies published between 2002-2022 either in English or German. Grey literature was included to consider possible reports and/or theses or dissertations that have not been published or peer-reviewed, but present original data. We included only studies with at least 50% of MA among the investigated study population (see Annex 1 with review protocol amendments).

Our review includes studies capturing either psychosocial working conditions and/or mental health among MA, regardless of whether these concepts constituted the primary focus of the respective studies. Our search strategy employed terms that capture theoretical constructs seeking to assess psychosocial working conditions (e.g., job-demands-control) and terminology related to them (e.g. stressor or resource) in order to increase sensitivity. Our search also included the term job satisfaction, because instruments used to capture it usually assess various domains pertaining to psychosocial working conditions. As for mental health, we were interested in common mental health disorders associated with psychosocial working conditions, e.g. anxiety disorder, burnout, and depression [24–27].

Due to the regional specificity of the profession, we only included studies conducted among MA in Germany. The identification of these studies is complicated due to the inconsistent translation of the term MA into English, hence for our population we used terms that have been commonly used in translations, but do not necessarily address the MA profession only (e.g., “medical assistant”).

### Information sources and search

We selected four databases after consultation with an information specialist (see acknowledgements), those were Medline (via PubMed), CCMed (a database for German sources, consulted via LIVIVO), Scopus and Google Scholar. All database searches were executed on October 10, 2022. We additionally conducted manual reference checking of the sources included after full text screening, and consulted with experts in the field (see acknowledgements) in order to find further sources not identified through our search strategy.

We designed the search strategies for the different databases in consultation with an information specialist. We tested different combinations of search terms, keywords, operators and wildcards reflecting our PCC-Framework and applied the combinations yielding the most relevant results. Annex 2 presents the full electronic search strategy used for PubMed (Medline). The remaining full text search strategies are shown in the review protocol (https://osf.io/8sev4).

We searched Google Scholar using German-language search terms in order to increase the likelihood to identify grey literature (e.g., theses from German universities or reports from German organizations). For Google Scholar, we followed the recommendation to only consider the first 200 references [28]. The Google Scholar search was conducted from a computer located in the city of Düsseldorf, Germany.

### Selection of sources of evidence and data charting process

After removal of duplicates in the citation management software, the remaining references were exported into the online software Rayyan.ai, where a second automatic duplicate search was performed. The screening of the remaining titles and abstracts was conducted simultaneously by KS and by a team composed of VM, AD and AL. Disagreements were solved through discussion. Full-text screening was conducted in parallel by two reviewers (KS and VM). Disagreements were solved through discussion and the involvement of a third reviewer (either AD or AL). In cases where more than one exclusion criteria applied, we recorded only the first criterion, following the order of exclusion criteria from the review protocol (https://osf.io/8sev4). Literature suggestions by experts and titles found through manual search underwent the same screening process.

We developed a data extraction sheet based on our research questions and established recommendations [21]. Two reviewers (KS and VM) piloted the extraction sheet making minor adjustments (see annex 1). We charted the following data: Reviewer, date, title, author(s) of publication, year of publication, name of journal/publication type (if not a journal article), aims/purpose, sample size, defining characteristics of study participants/setting and context related information, type of study (e.g. qualitative, quantitative, mixed methods), concepts used to capture psychosocial working conditions and/or mental health (if applicable), instruments used to capture psychosocial working conditions and/or mental health (if applicable), outcomes of significance to our concepts of interest, further findings of interest, authors’ conclusion/key findings (if applicable), and reviewer’s comments. After extraction of information from all sources, the charted results of both reviewers (KS and VM) were compared for accuracy and completeness.

As common in scoping reviews, we did not conduct a critical appraisal of individual sources due to the heterogeneity of the sources included.

### Synthesis of results

For quantitative studies presenting outcomes with mean values, we defined the mid-point of each scale as a cut-off for critical psychosocial working conditions or critical mental health outcomes. Depending on the direction of the respective scales (i.e., a lower value indicating unfavorable psychosocial working conditions or mental health, or vice-versa), mean values were rated as critical if these exceeded the cut-off or if they fell below the cut-off. This approach allowed us to compare studies reporting results with mean values on the same constructs. Studies presenting outcomes without mean values (all qualitative and *n* = 6 quantitative studies) were only summarized narratively.

For the narrative summary of psychosocial working conditions, we employed a deductive structure for patterns found based on several established theories aiming at capturing psychosocial working conditions: the Job-Demands-Control [29], the Job-Demands-Resources [30], and the Effort-Reward Imbalance [31] models. Job demands are broadly defined as sources of stress present in the work environment [29], and more specifically as “[…] aspects of the job that require sustained physical and/or psychological effort and are therefore associated with certain physiological and/or psychological costs” [30]. Job control includes aspects related to decision latitude [29] and predictability, entailing the objective and subjective evaluation of an individual’s control of the work situation as well as beliefs about it [32]. Job resources have been defined as aspects of the job that are either functional in achieving work goals, reduce job demands and the costs associated with them, or stimulate personal growth, learning, and development [33], whereas the theoretical framework of the Effort-Reward-Imbalance model recognizes three basic forms of job rewards: financial, status-related, and socioemotional [31]. We also included the category job satisfaction, as it may be viewed as a summary measure of psychosocial working conditions that are relevant to a given individual.

## Results

**Fig. 1:**
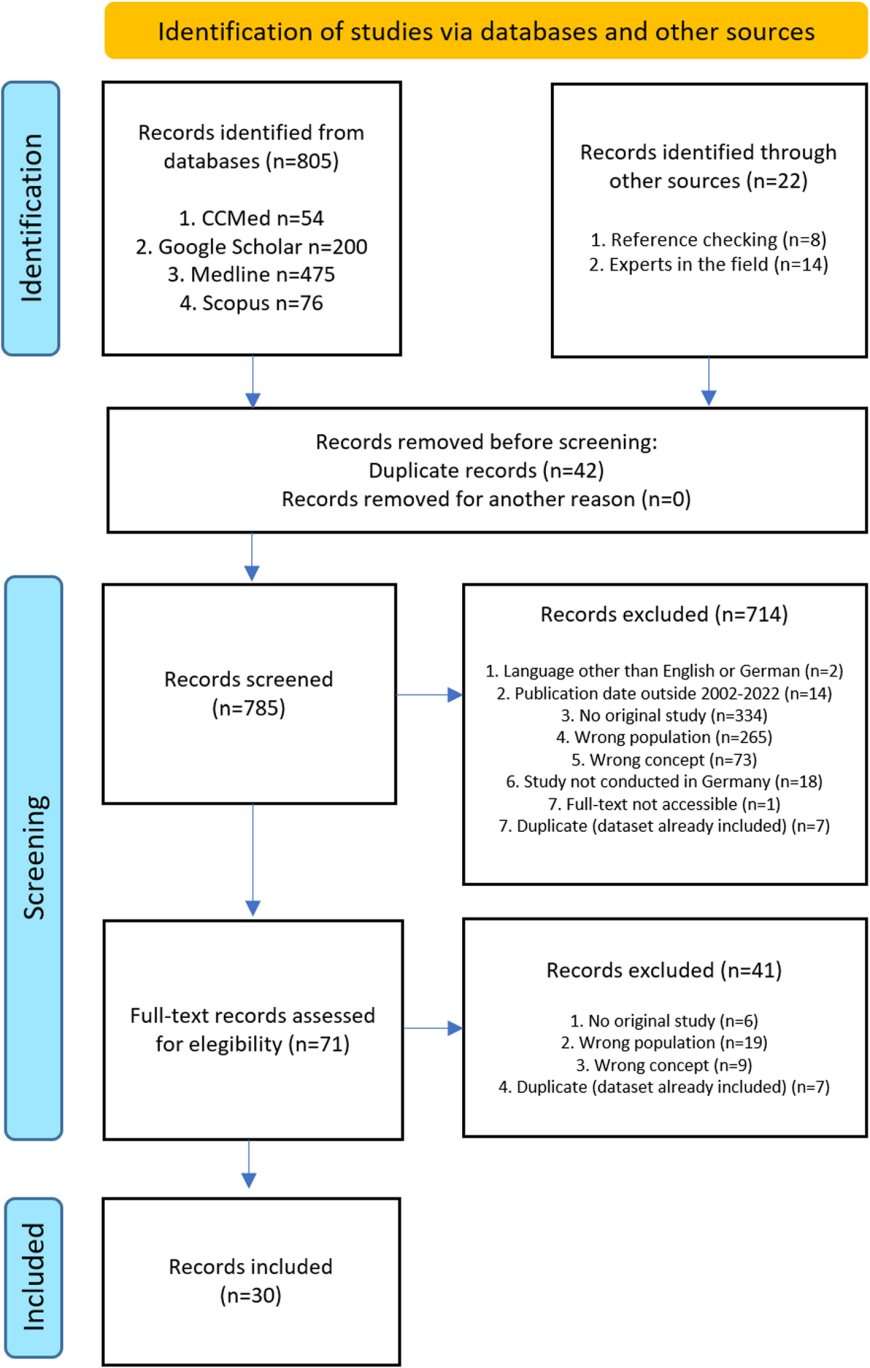
Flow diagram of excluded and included studies

Our systematic search of databases generated *n* = 805 hits (*n* = 54 for CCMed, *n* = 2,170 for Google Scholar, of which we only considered the first 200 hits, *n* = 475 for Medline, and *n* = 76 for Scopus). Additionally, we assessed *n* = 8 records found through manual reference checking and *n* = 14 identified through consultation with experts in the field. The detailed results of the search and screening process, including the reasons for exclusion at each stage, are illustrated in Fig. 1. After full-text screening, *n* = 30 sources were included in our scoping review, consisting of ten qualitative, 19 quantitative and one mixed methods study (see Table 1 for study characteristics).

**Table 1:** Overview of the characteristics of the included studies (*n* = 30)

Characteristics	N	%
Study type		
Qualitative	10	30.00
Quantitative	19	63.33
Mixed methods	1	3.33
Year of publication		
2002–2006	1	3.33
2007–2011	5	16.66
2012–2016	6	20.00
2017–2021	15	50.00
2022	3	10.00
Publication type		
Journal article, peer reviewed	26	86.66
Thesis or dissertation	3	10.00
Study report	1	3.33
Relevant concepts captured in study^a^		
Psychosocial working conditions	28	93.33
Mental health	8	26.66
MA population in study		
Only MA	19	63.33
At least 50% of study population MA	11	36.66

^a^Capturing both concepts is possible

The main characteristics of the selected sources are presented in Tables 1, 2, 3 and 4. Tables 2, 3 and 4 summarize the relevant results (concepts, instruments and outcomes) charted for each source of reference. Data such as defining characteristics of study participants/setting and context-related information were presented very heterogeneously or were not available in the different studies, making comparisons difficult. For example, some studies specified setting and context by mentioning if MA worked in general or specialized clinics [34, 35], whereas others studies reported the percentage of surveyed clinics located in rural or urban areas [36, 37] or if they were located in small towns, small or big cities [38]. Thus, we did not include these data in the summary tables. Our results suggest that studies reporting psychosocial working conditions and/or mental health among MA have increased substantially in the past years: the earliest study examining mental health among MA was published in 2014, and more than half of the examined studies were published within the last six years.

**Table 2:** Quantitative and mixed methods studies capturing psychosocial working conditions among medical assistants in Germany (2002–2022)

*Reference nr*	*Authors*	*Publi- cation year*	*n MA*^a^	*Study design*	*Aim/purpose of the study*	*Concepts used to capture psychosocial working conditions*	*Instruments used to capture psychosocial working conditions*	*Main results reported with potential score range (if relevant) and observed value*	*Critical cut-off*^b^
[39]	Degen et al.	2021	254	Quantitative, cross-sectional	To report the baseline characteristics of participants of an intervention study, focusing on job satisfaction and perceived chronic stress			*Scale transformed by authors to a score from 0 (“not satisfied at all”) to 100 (“fully satisfied”); mean (SD*^d^*)*	
						Job satisfaction	COPSOQ B11c^c^	72.58 (14.42)	No
[10]	Dormann et al.	2003	351	Quantitative, cross-sectional	To examine concepts of organizational and personal customer orientation and their (extended) customer service foundations as well as associations between them and customer-oriented control			*Scale range from 0 (“does not apply”) to 5 (“applies completely”); mean (SD)*	
						Decision latitude	ISTA6.0^e^	3.76 (0.70)	No
						Customer-oriented decision latitude	Self-developed instrument	3.63 (0.78) Scale transformed by authors to a score from 0 (“not satisfied at all”) to 100 (“fully satisfied”); mean (SDd)	No
						Work satisfaction	Kunin-item	5.52 (0.95)	No
[40]	Dreher et al.	2021	2150	Quantitative, cross-sectional	To investigate the prevalence of attitudes, stressors and work-related outcomes related to 2020 SARS-CoV-2 outbreak among MA working in inpatient and outpatient settings and to identify potential determinants of those outcomes			*Agree, n (%)*	Not calculated for any of the values (no mean value reported)
						SARS-CoV-2 attitudes	Self-developed instrument	a) The risk of contracting SARS-CoV-2 is higher for me than for a person of same age and sex from the general population: 1770 (82.3%)	
								b) I feel sufficiently informed about dealing with SARS-CoV-2 patients by my employer: 1428 (66.4%)	
								c) I feel sufficiently prepared for dealing with SARS-CoV-2 patients by my employer: 1301 (60.5%)	
								d) My workload has increased due to the SARS-CoV-2 pandemic: 1076 (50.0%)	
								e) I can use materials for personal protection at my work so that I feel sufficiently protected from contracting SARS-CoV-2: 702 (32.7%)	
						SARS-CoV-2 stressors		a) I am burdened by a feeling of not being able to let patients down during the crisis: 1630 (75.8%)	
								b) I am burdened by uncertainty about my financial situation during the crisis: 1448 (67.3%)	
								c) I am burdened with thoughts of a possible infection with SARS-CoV-2 during work hours: 1413 (65.7%)	
								d) I am burdened by the crisis-related shortfall of colleagues/staff at work: 1153 (53.6%)	
						SARS-CoV-2 work outcomes		a) At my work all necessary materials for personal protection from SARS-CoV-2 are sufficiently available for me: 516 (24.0%)	
[41]	Erler et al.	2012	15	Quantitative, experimental	To describe the effects of an intervention on work satisfaction and burnout risk in the explorative evaluation of that intervention			*Scale range apparently transformed by authors to a score from 0 (“fully satisfied”) to 100 (“not satisfied at all”); mean*^f^	
						Job demands	COPSOQ	a) Quantitative demands: 62.05	Yes
								b) Emotional demands: 44.05	No
								c) Demands for hiding emotions: 49.11	No
								d) Work-privacy conflict: 38.21	No
								*Scale range apparently transformed by authors tor a score from 0 (“not satisfied at all”) to 100 (“fully satisfied”); mean*	
						Influence and development opportunities		a) Influence at work: 32.14	Yes
								b) Degree of freedom at work: 23.66	Yes
								c) Possibilities for development: 72.32	No
								d) Meaning of work: 87.5	No
								e) Commitment to workplace: 71.43	No
						Interpersonal relations and leadership		a) Predictability: 68.75	No
								b) Role clarity: 86.61	No
								c) Quality of leadership: 65,63	No
								d) Social support: 73.66	No
								e) Feedback: 41.07	Yes
								f) Social relations: 61.61	No
								g) Sense of community: 80.36	No
								*Scale range apparently transformed by authors to a score from 0 (“fully satisfied”) to 100 (“not satisfied at all”); mean*	
								h) Role conflicts: 34.82	No
								i) Bullying: 19.64	No
						Job insecurity		a) Job insecurity: 26.34	No
								*Scale range apparently transformed by authors to a score from 0 (“not satisfied at all”) to 100 (“fully satisfied”); mean*	
						Job satisfaction		a) Job satisfaction: 73.03	No
[42]	Fauser et al.	2020	1438	Quantitative, cross-sectional	To determine the predictive value of the dimensions of the ERI^g^ model for the construct burnout in a sample of MA in Germany			*Score range: 3–12, higher scores indicating higher effort; mean (SD*)	
						Effort-reward imbalance	ERI (short version)	a) Effort: 10.4 (1.38)	Yes
								*Score range: 6–27, higher scores indicating higher rewards; mean (SD)*	
								b) Reward: 14.3 (2.91)	Yes
								*Score > 1 indicates effort-reward imbalance; mean (SD)*	
								c) ERI-Ratio: 1.54 (0.43)	Yes^h^
								*Score range: 4–16, higher scores indicating higher overcommitment; mean (SD)*	
						Over-commitment		a) Overcommitment: 10.8 (2.83)	Yes
[36]	Feindel et al.	2019	12	Mixed-methods, cross-sectional	To develop and pilot test a questionnaire evaluating MA attitudes towards task shifting and their perceptions of its challenges, and to assess the psychometric properties of that questionnaire			*Scale range from 1 to 7, higher value indicating higher satisfaction; median (IQR*^j^*):*	Not calculated for any of the values (no mean value reported)
						Working conditions and job satisfaction	Self-developed instrument and WCW^i^	a) Freedom of working method: 5 (3–6)	
								b) Colleagues and fellow workers: 6 (5–6)	
								c) Recognition for work: 4 (2–6)	
								d) Amount of responsibility: 5 (4–6)	
								e) Income: 3 (1–3)	
								f) Opportunity to use abilities: 5 (4–6)	
								g) Hours of work: 5 (3–6)	
								h) Amount of variety in job: 5 (4–6)	
								i) Mental working conditions: 4 (3–5)	
								j) Overall satisfaction: 5 (4–6)	
						Concerns regarding delegation	Self-developed instrument	a) Did not see any financial incentive from the additional qualification: *n* = 98 (35.8%)	
								b) Felt tasks were not sufficiently defined: *n* = 95 (34.7%)	
								c) Feared a lack of acceptance by patients: *n* = 79 (28.8%)	
								d) Reported that it was unclear who bore responsibility and was therefore liable: *n* = 74 (27%)	
								e) Feared that competition with other practice assistants could arise: *n* = 52 (19%)	
								f) Stated that their remuneration had increased because of an additional qualification: *n* = 68 (24.8%)	
[43]	Gavartina et al.	2013	586	Quantitative, cross-sectional	To evaluate the job satisfaction and organizational attributes of practice assistants in general practices in Germany and to explore associations between them			*Scale range from 1 = “extreme dissatisfaction” to 7 = “extreme satisfaction”; mean (SD)*	
						Job satisfaction	WCW	a) Physical working condition: 5.18 (1.32)	No
								b) Freedom of working method: 5.20 (1.35)	No
								c) Colleagues and fellow workers: 5.87 (1.28)	No
								d) Recognition for work: 5.07 (1.47)	No
								e) Amount of responsibility 5.38 (1.33)	No
								f) Income 3.89 (1.79)	Yes
								g) Opportunity to use abilities 5.26 (1.26)	No
								h) Hours of work 5.34 (1.49)	No
								i) Amount of variety in job 5.49 (1.22)	No
								j) Overall job satisfaction 5.74 (1.19)	No
								*Scale range from 1 = “fully disagree” to 5 = “fully agree”, according to the number of items in the respective scales, the potential score range cannot be determined. We assume that the score was divided by the number of items, with a higher value indicating better organizational attributes; mean (SD)*	
						Organizational attributes for primary care	SOAPC^k^	a) Communication: 3.92 (0.48)	No
								b) Decision making: 3.95 (0.64)	No
								c) Stress: 3.53 (0.73)	Yes
								d) Change [changes in the work organization and teamwork]: 3.13 (0.79)	No
								e) Overall score: 3.70 (0.48)	No
[44]	Göbel et al.	2022	254^l^	Quantitative, cross-sectional	To analyze the relationship between work-privacy-conflict and job satisfaction among German general practitioners and MA			*Scale range transformed by authors to a numerical scale from 0 to 100, higher score indicating a stronger work-privacy conflict; mean (SD)*	
						Work-privacy conflict	COPSOQ	a) Work-privacy-conflict: 32.67 (28.35)	No
[45]	Goetz et al.	2011	2332^m^	Quantitative, cross-sectional	To evaluate the job satisfaction of German general practitioners and their non-physician staff			*Scale range from 1 = “extreme dissatisfaction” to 7 = “extreme satisfaction”; mean (SD)*^*n*^	
						Job satisfaction	WCW	a) Freedom of working method: 5.82 (1.23)	No
								b) Colleagues and fellow workers: 6.18 (1.02)	No
								c) Recognition for work: 5.41 (1.49)	No
								d) Amount of responsibility: 5.92 (1.34)	No
								e) Income: 4.79 (1.65)	No
								f) Opportunity to use abilities: 5.82 (1.17)	No
								g) Hours of work: 5.75 (1.32)	No
								h) Amount of variety in job: 5.94 (1.15)	No
								i) Physical working condition: 5.63 (1.25)	No
								j) Overall job satisfaction: 5.95 (1.05)	No
[37]	Goetz et al.	2013	1158	Quantitative, cross-sectional	To evaluate the job satisfaction of MA in German general practice and explore the associations between job satisfaction, staff characteristics and organizational culture within the practice			*Scale range from 1 = “extreme dissatisfaction” to 7 = “extreme satisfaction”; mean (SD)*	
						Job satisfaction	WCW	a) Freedom of working method: 5.71 (1.22)	No
								b) Colleagues and fellow workers: 6.13 (1.03)	No
								c) Recognition for work: 5.33 (1.52)	No
								d) Amount of responsibility: 5.84 (1.11)	No
								e) Income: 4.62 (1.68)	No
								f) Opportunity to use abilities: 5.77 (1.17)	No
								g) Hours of work: 5.72 (1.39)	No
								h) Amount of variety in job: 5.94 (1.10)	No
								i) Physical working condition: 5.51 (1.28)	No
								j) Overall job satisfaction: 5.84 (1.09)	No
								*Scale range from 1 (“fully disagree”) to 5 (“fully agree”), higher score indicating better organizational culture; mean (SD)*	
						Organizational culture	EPA^o^	a) Responsibilities within the practice team are clear: 4.26 (0.88)	No
								b) Offering suggestions for improvement: 4.09 (1.06)	No
								c) Suggestions for improvement are taken seriously: 3.91 (1.10)	No
								d) Working atmosphere in the practice team is good: 4.19 (0.97)	No
[46]	Hoffmann et al.	2020	550	Quantitative, cross-sectional	To assess the mental workload of MA working in German primary care practices, to identify resources and stressors and to compare the results with aggregate data from 23 professions			*Scale range from 1 (“does not apply at all”) to 5 (“is completely true”), high scores (> 3) are considered positive; mean (95% CI*^q^*)*	
						Job content	KFZA^p^	a) Versatility: 3.6 (3.58–3.70)	No
								b) Completeness of task: 3.5 (3.41–3.57)	No
								*Scale range from 1 = “does not apply at all” to 5 = “is completely true”, high scores (> 3) are considered positive; mean (95% CI)*	
						Resources	KZFA	a) Scope of action: 3.4 (3.37–3.49)	No
								b) Social support: 4.0 (3.98–4.12)	No
								c) Cooperation: 3.6 (3.53–3.66)	No
								*Scale range from 1 = “does not apply at all” to 5 = “is completely true”, high scores (> 3) are considered negative; mean (95% CI*^q^*)*	
						Stressors	KZFA	a) Qualitative work demands: 2.2 (2.14–2.29)	No
								b) Quantitative work demands: 2.9 (2.83–3.01)	No
								c) Work disruption: 2.7 (2.67–2.81)	No
								d) Workplace environment: 2.2 (2.13–2.30)	No
								*Scale range from 1 = “does not apply at all” to 5 = “is completely true”, high scores (> 3) are considered positive; mean (95% CI)*	
						Organizational culture	KZFA	a) Information and participation: 3.6 ( 3.57–3.73)	No
								b) Benefits: 2.9 (2.77–2.94)	Yes
[47]	Mahler et al.	2007	89	Quantitative, cross-sectional	To assess the frequency of participation in training courses as well as the reasons and obstacles for participation, and to find out which topics MA consider interesting and what effects they expect a training course to have on their work situation	Assessment of the own work situation	Self-developed instrument	Results provided through a bar-chart, exact values missing	Not calculated for any of the values (no mean value reported)
						Further training		*Only main answers reported here:*	
								a) Job-related training measures were taken by 62.5% of the physician assistants one to three times a year, more than 3 training courses a year even by about a quarter of the respondents (26.1%)	
								b) Reasons to participate in a training course based on reported frequency (main reasons mentioned): 1) To broaden expertise (*n* = 88; 100%); 2) To obtain suggestions for changes in the daily work environment (*n* = 71; 80.7%)	
								c) Obstacles to participation in training courses: 1) Training is too expensive (*n* = 26; 42.6%); 2) not compatible with the family situation (*n* = 24; 39.3%); 3) a lack of career opportunities (24.6%)	
								d) Offer on training courses for MA is sufficient or very good (*n* = 29; 33,3%)	
								e) Complained about the clarity of the continuing education programs (42.5%)	
								f) *n* = 77 (87.5%) MA receive support from their supervisor in attending continuing education courses (e.g. cover of participation costs and/or travel costs, payment of overtime and/or time off)	
[3]	Mergenthal et al.	2021	2371	Quantitative, cross-sectional	To answer how satisfied are MA^a^ with various aspects of their work, and which socio-demographic factors influence job satisfaction			*Scale range from 1 = “extreme dissatisfaction” to 7 = “extreme satisfaction”; mean (SD)*	
						Job satisfaction	WCW^i^	a) Psychological workload: 4.61 (1.7)	No
								b) Freedom of working method: 4.98 (1.7)	No
								c) Colleagues and fellow workers: 5.50 (1.7)	No
								d) Recognition for work: 4.76 (1.8)	No
								e) Amount of responsibility: 5.53 (1.4)	No
								f) Income: 3.73 (1.9)	Yes
								g) Opportunity to use abilities: 4.91 (1.6)	No
								h) Hours of work: 5.01 (1.8)	No
								i) Amount of variety in job: 5.01 (1.7)	No
								j) Overall job satisfaction: 5.20 (1.6)	No
[34]	Oelschlegel, H.	2007	77	Quantitative, cross-sectional	To research the role of MA in methadone substitution in GP^r^practices in a southern German region in order to make suggestions to improve their training	Work motivation	Self-developed instrument	Reasons for working motivation:	Not calculated for any of the values (no mean value reported)
								a) Earn money (*n* = 69): 31,9% (*n* = 22): very important, 33,3% (*n* = 23): important; 26,2% (*n* = 18): moderately important; 7,2% (*n* = 5): little important; *n* = 0: unimportant; 1,4% (*n* = 1): completely unimportant	
								b) Helping other people (*n* = 71): 53,5% (*n* = 38): very important; 35,2% (*n* = 25): important; 5,6% (*n* = 4): moderately important; 1,4% (*n* = 1): little important; 1,4% (*n* = 1): unimportant; 2,8% (*n* = 2): completely unimportant	
								c) Working in a team (*n* = 69): 31,9% (*n* = 22): very important; 50,7% (*n* = 35): important; 10,1% (*n* = 7): moderately important; 4,3%(*n* = 3): little important; 1,4%(*n* = 1): unimportant; 1,4% (*n* = 1): completely unimportant	
								d) Recognition by the boss (*n* = 69): 21,7% (*n* = 15): very important; 31,9% (*n* = 22): important; 20,3% (*n* = 14): moderately important; 14,5%(*n* = 10): little important; 5,8%(*n* = 4): unimportant; 5,8% (*n* = 4): completely unimportant	
								e) Recognition through patients or relatives (*n* = 70): 24,6% (*n* = 17): very important; 27,5% (*n* = 19): important; 20,3% (*n* = 14): moderately important; 15,9% (*n* = 11): little important; 4,3% (*n* = 3): unimportant; 8,7% (*n* = 2) completely unimportant	
								f) Social recognition (*n* = 69): 2,9% (*n* = 2): very important; 22,8% (*n* = 16): important; 12,8% (*n* = 9): moderately important; 17,1% (*n* = 12): little important; 20% (*n* = 14): unimportant; 22,9% (*n* = 16): completely unimportant	
								g) Personal experience (*n* = 67): 4,5% (*n* = 3): very important; 7,5% (*n* = 5): important; 16,4% (*n* = 11): moderately important; 10,4% (*n* = 7): little important; 4,5% (*n* = 3): unimportant; 56,7% (*n* = 38): completely unimportant	
						Job satisfaction		a) Overall job satisfaction (*n* = 77): 94% mostly satisfied vs. 6% mostly unsatisfied	
						Being afraid		a) Being afraid in the context of their work with substitution patients: 67,5% (*n* = 52): reported not to be afraid; 28,6% (*n* = 22): reported to be seldom afraid; 3,9% (*n* = 3): reported to be often afraid	
[35]	Scharf et al.	2019	887	Quantitative, cross-sectional	To quantify needs and desired improvements from a previous qualitative study as a starting point for the development, implementation and evaluation of interventions			*n (%):*	
						Needs regarding: Working conditions, work organization; rewards from supervisor, task-related independence, working climate, leadership	Self-developed instrument	a) I would like to have more responsibility in my job: 230 (26.3)	Not calculated for any of the values (no mean value reported)
								b) I would like to have greater scope of action and freedom of choice: 356 (40.9)	
								c) I would like to independently advise patients about their disease: 236 (27.1)	
								d) I would like to make home visits: 112 (12.9)	
								e) I would like to have more educational opportunities: 478 (54.9)	
								f) I would like to have additional breaks: 346 (39.9)	
								g) I would like to have different opening hours of the practice/clinic: 368 (42.3)	
								h) I would like to have more staff at my workplace: 488 (55.7)	
								i) I would like to work less hours: 466 (53.9)	
								j) I wish for more understanding from my supervisor: 528 (60.3)	
								k) I would like to have a better working climate: 400 (45.9)	
								l) I wished from improved interactions between colleagues: 330 (37.9)	
								m) I wish for more appreciation for my work from my supervisor: 531 (60.8)	
								n) I wish for more recognition for my work from the society: 654 (75.4)	
								o) I would like to have a higher salary: 759 (87.0)	
								p) I would like the physicians to have educational opportunities related to organizational leadership: 653 (75.1)	
								q) I would like to have less documentation in my day-to-day work: 659 (76.0)	
								r) I wish for a better organization of the practice/clinical procedures: 479 (54.7)	
								s) I would like to include the internet /new media in my daily work: 277 (31.8)	
								t) I would like to have less multitasking: 585 (67.5)	
[48]	Szecsenyi et al.	2011	3011^s^	Quantitative, cross-sectional	To evaluate whether there is an association between patient satisfaction and job satisfaction of the members of patient care teams			*Scale range from 1 = “extreme dissatisfaction” to 7 = “extreme satisfaction”; mean (95% CI*^q^*)*^t^	
						Job satisfaction	WCW^i^	a) Freedom of working method: 5.82 (5.77–5.86)	No
								b) Colleagues and fellow workers: 6.17 (6.14–6.21)	No
								c) Recognition of work: 5.42 (5.37–5.48)	No
								d) Amount of responsibility: 5.92 (5.87–5.96)	No
								e) Income: 4.77 (4.71–4.83)	No
								f) Opportunity to use abilities: 5.82 (5.77–5.86)	No
								g) Hours of work: 5.73 (5.68–5.78)	No
								h) Amount of variety in job: 5.93 (5.98–5.97)	No
								i) Physical working condition: 5.62 (5.57–5.67)	No
								j) Overall job satisfaction: 5.94 (5.90–5.98)	No
								In summary, non-physician staff rated the job satisfaction with mean = 5.71 (SD = 0.91)	No
								*Mean (SD)*	
						Estimation of the practice organization	Instrument of unspecified origin	Evaluation of the organization of the practice team: 4.09 (0.27)	No
[49]	Vu-Eickmann et al.	2018	887	Quantitative, cross-sectional	To examine the psychosocial working conditions of MA and possible associations with health outcomes, quality of care indicators and the intention to leave			*Value range 6–24; mean (SD)*	
						Effort-Reward imbalance	ERI^g^	a) Effort (cut-off ≥ 21): 18.56 (3.19)	Yes
								*Value range (11–44); mean (SD)*	
								b) Reward (cut-off ≥ 31): 28.25 (5.98)	No
								*Score > 1 indicates effort-reward imbalance; mean (SD)*	
						Work stress		c) ERI ratio (Effort*11/Reward*6): 1.28 (0.42)	Yes^u^
								d) Prevalence of work stress (according to ERI ratio): *n* = 616 (73.77%)	Not calculated
								*Mean (SD)*	
						MA-specific working conditions: Workload, job control, collaboration, gratification, practice organization, resources	Self-developed instrument	a) Workload (value range 6–24): 17.36 (4.19)	Yes
								b) Job control (value range 6–24): 21.11 (2.71)	Yes
								c) Collaboration (value range 4–16): 8.41 (2.85)	No
								d) Gratification crisis (value range 4–16): 11.52 (2.66)	Yes
								e) Practice organization (value range 3–12): 6.56 (2.08)	No
								f) Resources (value range 3–12): 4.63 (1.71)	No
								g) Supervisor (value range 3–12): 8.01 (2.35)	Yes
[50]	Zaroti, S.	2015	586	Quantitative, cross-sectional	To explore what psychosocial work stress general practitioners and MA are exposed to, the differences between GP and MA regarding their psychosocial working environment in terms of form of employment and gender, and associations between psychosocial stress and burnout	Job demands	COPSOQ^c^	*Scales were transformed to a range from 0 (“fully satisfied”) to 100 (“not satisfied at all”); mean (SD)*	
								a) Quantitative demands: 49.19 (16.78)	No
								b) Emotional demands: 47.87 (19.12)	No
								c) Demand to hide emotions: 44.88 (23.10)	No
								d) Work family privacy conflict: 25.41 (24.44)	No
								*Scales were transformed to a range from 0 (“not satisfied at all”) to 100 (“fully satisfied”); mean (SD)*	
						Influence and development opportunities	COPSOQ	a) Influence at work: 41.16 (21.41)	Yes
								b) Decision latitude: 42.67 (20.03)	Yes
								c) Possibilities for development: 69.05 (14.47)	No
								d) Meaning of work: 83.75 (15.18)	No
								e) Commitment to workplace: 63.89 (17.60)	No
						Social relations and leadership	COPSOQ	a) Predictability: 67.03 (20.82)	No
								b) Role clarity: 81.55 (13.69)	No
								c) Quality of leadership: 65.85 (20.90)	No
								d) Social support: 78.23 (18.50)	No
								e) Feedback: 51.39 (23.10)	No
								f) Social relations: 42.29 (16.88)	Yes
								g) Sense of community: 85.92 (15.90)	No
								*Scales were transformed to a range from 0 (“fully satisfied”) to 100 (“not satisfied at all”); mean (SD)*	
						Insecurity at workplace	COPSOQ^c^	h) Bullying: 17.64 (22.18)	No
								a) Job insecurity: 20.20 (17.97)	No
								*Scale was transformed to a range from 0 (“fully satisfied”) to 100 (“not satisfied at all”); mean (SD)*	
						Job satisfaction	COPSOQ	a) Job satisfaction: 73.61 (14.10)	No

^a^Medical assistants

^b^For studies presenting mean values, we defined the mid-point of each scale as a cut-off for critical psychosocial working conditions outcomes. Depending on the direction of the respective scales (i.e., a lower value indicating unfavorable psychosocial working conditions, or vice-versa), mean values were rated as critical if these exceeded the cut-off

^c^Copenhagen Psychosocial Questionnaire

^d^Standard deviation

^e^Instrument for the stress-related task analysis

^f^All mean values from this study were derived from pre-intervention assessment

^g^Effort-reward imbalance

^h^Based on own cut-off value for the scale, not on our calculations

^i^Warr-Cook-Wall job satisfaction scale

^j^Interquartile range

^k^Survey of Organizational Attributes for Primary Care

^l^*N* = 254 MA from a total sample of *n* = 366 including also *n* = 84 practice owners and *n* = 28 employed physicians

^m^*N* = *2,332 (81.0%) MA out of n* = *2,878 non-physician staff members included in the study*

^n^All values for non-physician staff

^o^European Practice Assessment

^p^Short Questionnaire for Job Analysis (German: *Kurz-Fragebogen zur Arbeitsanalyse*

^q^Confidence interval

^r^General practitioners

^s^*N* = *3011 non-physician staff in primary practices (most probably medical assistants)*

^t^All results for non-physician staff

^u^According to the scale’s own cut-off value

**Table 3:** Qualitative studies capturing psychosocial working conditions among medical assistants in Germany (2002–2022)

*Reference nr*	*Authors*	*Publication year*	*n MA*^a^	*Study design; data collection method*	*Aim/purpose of the study*	*Concepts capturing psychosocial working conditions*	*Main outcomes/Key findings*
[51]	Ehlers-Mondorf et al.	*2021*	34	Qualitative, cross-sectional; semi-structured interviews	To present the experience of MA with the SARS-CoV-2 pandemic and address their suggestions for improvement of pandemic preparation	Teamwork	More workload and stressors for MA due to the SARS-CoV-2 pandemic. MA felt that their role as first contact persons for patients during the pandemic did not receive sufficient recognition
						Billing issues	
						Unstructured information flow	
						Lack of appreciation	
[52]	Gensinchen et al.	2009	26	Qualitative, cross-sectional; semi-structured interviews	To describe the perceptions and experiences of MA who provided case management to patients with depression in small primary care practices	Role perception	MA’s role as case managers was perceived as personally and professionally enriching. Integrating case management in daily work was difficult for many MA because of insufficient time, unexpected patient-related factors, and lack of understanding by colleagues and supervisors. Relationships with patients were especially important for MA and could be both a burdening and a relieving factor. The interaction with depressive patients was described as difficult and demanding. General practitioners support or lack thereof influenced the perceived stress. Incomplete knowledge on depression increased strain
						Burdening and relieving factors	
						Interaction with depressed patients	
						Collaboration with doctor	
						Disease conception	
[53]	Hoffmann et al.	2022	12^v^	Qualitative, cross-sectional; semi-structured interviews	To present the qualitative results from the process evaluation of an intervention for COVID-19 surveillance and care for COVID-19 patients with increased risk used by VERAHs (specially qualified MA) and general practitioners	Sense of security	The implementation of the intervention gave MA a sense of security and support. MA emphasized additional workload as particularly negative because the intervention added additional tasks and strain in the already tightly organized daily practice. Fear for additional workload and overtime was uttered before the intervention was implemented. Some MA were overwhelmed by the workload during the pandemic, the time required to implement the intervention was considered a major problem
						Workload and overtime	
						Job satisfaction	
[54]	Kathmann et al.	2013	10	Qualitative, cross-sectional; semi-structured interviews with MA^a^, expert interviews	To explore the degree of precarity of the MA job through an examination of working conditions	Precarious work	Low salary most central aspect in the precarity of the MA profession. MA dissatisfied with their low salary (main reason to quit the profession). Comparison with other professions (doctors, nurses) in terms of salary and social standing/ recognition leads to disappointment and dissatisfaction, also with their supervisors/employers. Overtime is considered to have a strong impact on private life, restricting MA’s social sphere and leisure time and leading to desire to have flexible working hours. MA reported a limited scope of action regarding negotiation of working hours and salary. Further qualifications considered to barely have an impact on salary or career prospects
[38]	Mergenthal et al.	2012	6	Qualitative, cross-sectional; semi-structured interviews	To explore the role of MA with a migration background in the general practices	Setting GP^r^practice (e.g. work and role distribution)	MA with migration background experience ad-hoc interruptions to take over unplanned activities such as translating. They assume the role of translators and cultural mediators. Help or translation is often requested by patients or physicians. The interviewed MA felt integrated into the family practice setting and reported no additional stress or resources emerging from the additional tasks they were assigned due to their migration background
						Communication and interaction with patients	
						Language connection	
[55]	Preiser et al.	2021	19^w^	Qualitative, cross-sectional; participant observation, semi-structured interviews and focus group discussions	To answer how GP fulfill their role as entrepreneurs and leaders responsible for the occupational safety of their employees regarding the organization of working time, and what psychosocial demands and resources result from the way how working time for practice teams is organized	Work content and task	“Unplannable” events considered part of the daily routine during consultation hours and lead to perceived psychological stress for GP and the practice team. MA expressed satisfaction with the flexibility of different working time models and the GP’s overall willingness to adjust working time models and hours to their needs. The immediate treatment of patients was favored over predictable working times for MA and GP, as a result taking lunch breaks or finishing work on time was raised as challenging by MA. MA rarely took mini-breaks, the authors associated this observation not with a high workload, but with the design of the workplace. The practice staff described mutual social support when planning individual vacations
						Organization of work	
						Working environment	
						New forms of work	
[56]	Rothe, M.	2019		Qualitative (partly quantified), cross-sectional; semi-structured interviews	To examine the role understanding, perceptions, feelings and scope of action of MA dealing with “difficult” and psychosomatic ill patients in general practices	Stressors and facilitators	MA reported stress due to high workload, high time pressure, a high amount of administrative work and confidentiality. The behavior of colleagues, human resources planning complicating work (e.g. sick leave) and behavior of some patients were also considered stressors. A lower workload, structured work, support from supervisor, collegiality and nice patients were considered facilitators. High satisfaction (quantified) was reported for support from colleagues and support from supervisor. The latter was considered to be very important
						Perceived support by supervisor and team	
[57]	Tasrouha et al.	2020	1920	Qualitative, cross-sectional; participant observation and focus group discussions	To gain an in-depth understanding of psychosocial demands and resources in the primary care setting	Work content and tasks	The key psychological demands observed were incomplete execution of tasks, frequent interruptions, high levels of work intensity, simultaneous processing of several tasks, and tightly coupled work processes. Also noise, missing/ unsuitable/unused/incorrectly used equipment/software and the feeling of being under constant observation were important demands. Key resources mentioned were an appropriate scope of action though influence on the sequence of activities and sufficient patient-related information. The possibility to take mini-breaks and efficient communication and cooperation within the team (e.g. clearly defined responsibility areas in the laboratory) were important resources. A positively perceived teamwork and a supportive working environment (e.g. access to suitable workstations, equipment and software) were further reported as important resources
						Organization of work	
						Working environment	
						New forms of work	
[58]	Vu-Eickmann et al.	2017	26	Qualitative, cross-sectional; semi-structured interviews	To gain in-depth insights into MA^a^ work stress and resources, as well as prevention options for intervention needs	Key stressors	The perceived job-related stress is overall very high. Very quiet periods at work alternate with work-intensive periods. Factors contributing to (high) workload: high patient volume with a simultaneous shortage of staff, increased documentation effort, inefficient practice organization, inability to take breaks (leading to physical and mental strain), considerable overtime, which is not compensated. Unforeseeable events lead to low job control. These arise from frequent interruptions of work processes and the requirement of multi-tasking (e.g. through phone calls, physicians concerns or emergency patients, technical problems and missing materials). Collaboration can be a stressor (e.g. supervisor who interrupts work process of MA, unpredictable or emotionally short-tempered employer, lack of support within the team, negative change in patient attitudes and expectations) or a resource (positive patient interaction, interaction with colleagues and social support, a supporting supervisor). Everyday work (broad and varied range of activities, a certain scope of action in the own core areas) is considered a key resource. Desired improvement needs include: higher salary, greater recognition (by society, patients and supervisors), improvement of the staff: patient ratio, more regulated working hours, reduced documentation, lower hierarchical structure (with supervisor), continuing education and training opportunities for physicians (organizational leadership)
						Key resources	
						Desired improvement needs	
[59]	Werdecker et al.	2022	15	Qualitative, cross-sectional; semi-structured interviews and observations	To explore what contributes to the feeling of happiness among general practitioners’and their staff in the work context	Teamwork	Teamwork was observed to be important for physicians and staff in terms of work satisfaction, collegial support, broad scope of action to complete tasks are considered important for teamwork. Laughing together, trust shown for the design of one’s own working area in the non-physician team (e.g. laboratory, registration desk) was perceived as appreciative and contributed to a feeling of happiness. Team meetings were perceived as valuable instruments for strengthening collegial cooperation, as well as exchange within the team to handle difficult and stressful situations. Long-lasting intensive relations with patients and fitting between patient and team seemed to contribute to harmony in daily work. Feedback from patients on the effectiveness or the observation of a positive development as well as gratitude from patients also considered to contribute to the happiness and satisfaction of MA^a^at work
						Relationship with patients	
						Patient fit	
						Effectiveness of one’s own actions, success and recognition	

^a^Medical assistants

^r^General practitioners

^v^*N* = 12 VERAHs (MA with special training as care assistants in the family practice)

^w^Only information from practice teams (*n* = 19 MA) was included in this summary

**Table 4:** Characteristics and findings of studies capturing mental health among medical assistants in Germany (2002–2022)

*Refe-rence nr*	*Authors*	*Publi-cation year*	*n MA*^a^	*Study design*	*Aim/purpose of the study*	*Concepts used to capture mental health*	*Instru-ments used to capture mental health*	*Main results reported with potential score (if relevant) and value*	*Critical cut-off*^x^
[39]	Degen et al.	2021	250	Quantitative, cross-sectional	To report the baseline characteristics of participants of an intervention study, focusing on job satisfaction and perceived chronic stress			*Value range 0–48, with higher scores indicating higher levels of perceived stress in the past 3 months; mean (SD*^d^*)*	
						Chronic stress	TICS-SSCS^y^	19.62 (9.07)	No
[40]	Dreher et al.	2021	2150	Quantitative, cross-sectional	To investigate the prevalence of attitudes, stressors and work-related outcomes related to 2020 SARS-CoV-2 outbreak among MA working in inpatient and outpatient settings and to identify potential determinants of those outcomes			*Reported as prevalence of a positive screening*	
						Depression	PHQ-2^z^	29.9%	Not calculated for prevalences
						Generalized anxiety disorder	GAD-2^aa^	42.6%	
[41]	Erler et al.	2012	15	Quantitative, longitudinal	To describe the effects of an intervention on work satisfaction and burnout risk in the explorative evaluation of that intervention			*Value range 0–100, with higher scores indicating higher exhaustion*^ac^*; mean*	
						Burnout	CBI^ab^	44.64	No
						Cognitive stress symptoms	COPSOQ^c^	27.68	No
[42]	Fauser et al.	2020	1438	Quantitative, cross-sectional	To determine the predictive value of the dimensions of the ERI model for the construct burnout in a sample of MA in Germany			*Value transformed into range 0–100, with higher scores indicating higher exhaustion; mean (SD*^d^*)*	
						Burnout	CBI	57.2 (20.05)	Yes
[60]	Karlsen et al.	2021	40	Quantitative, cross-sectional	To evaluate the differential stress inventory (DSI) by evaluating the objective and subjective stress differences in the five DSI types in the occupational setting			*Reported as n, MA categorized into DSI types*	
						Stress	DSI^ad^	1) Normal: 21	Not calculated for any of the values
								2) Overstressed: 4	
								3) Stress resistant: 8	
								4) Low stress/high coping: 4	
								5) High stress/high coping: 3	
[61]	Viehmann et al.	2017	550	Quantitative, cross-sectional	To analyze the prevalence of chronic stress for general practitioners and MA			*Reported as median (IQR*^j^*)*	
						Chronic stress	TICS-SSCS^y^	16 (12.25)	Not calculated for any of the values
								*Applying DEGS1*^ae^ c*ut-off for high stress (TICS-SSCS ≥ 23), n (%)*	
						Prevalence of high strain among MA due to chronic stress		550 (26.4)	
[49]	Vu-Eickmann et al.	2018	887	Quantitative, cross-sectional	To examine the psychosocial working conditions of MA and possible associations with health outcomes, quality of care indicators and the intention to leave			*Mean (SD)*	
						Depressiveness	PHQ-2	1.56 (1.46)	No
						Anxiety	GAD-2	1.47 (1.66)	No
								*n (%)*	
						Prevalence of depressiveness	PHQ-2	153 (17.45)	Not calculated for prevalences
						Prevalence of anxiety	GAD-2^aa^	177 (20.14)	
[50]	Zaroti, S.	*2015*	586	Quantitative, cross-sectional	To explore what psychosocial work stress are GP and MA exposed to, the differences between GP and MA regarding their psychosocial working environment in terms of form of employment and gender, and associations between psychosocial stress and burnout			*Mean (SD*^d^*)*^af^	
						Burnout	COPSOQ^c^	38.02 (19.81)	No
						Cognitive stress symptoms	COPSOQ	26.36 (18.18)	No

^a^Medical assistants

^c^Copenhagen Psychosocial Questionnaire

^d^Standard deviation

^j^Interquartile range

^x^For studies presenting mean values, we defined the mid-point of each scale as a cut-off for mental health outcomes. Depending on the direction of the respective scales (i.e., a lower value indicating unfavorable mental health, or vice-versa), mean values were rated as critical if these exceeded the cut-off

^y^Trier Inventory for Chronic Stress

^z^Patient Health Questionnaire (short version)

^aa^Generalized Anxiety Disorder questionnaire

^ab^Copenhagen Burnout Inventory

^ac^All mean values from this study are derived from pre-intervention assessments

^ad^Differential Stress Inventory

^ae^German Health Interview and Examination Survey for Adults (German: *Studie zur Gesundheit Erwachsener in Deutschland*

^af^All values for the MA group (*n* = 586) only

### Synthesis of results

#### Psychosocial working conditions of medical assistants in quantitative and mixed methods studies

Table 2 presents a summary of quantitative and mixed methods studies capturing psychosocial working conditions with the respective concepts, instruments and outcomes of interest.

##### Job satisfaction

Job satisfaction was the most commonly captured concept related to psychosocial working conditions among MA (*n* = 10 quantitative and mixed methods studies). These studies either focused on evaluating job satisfaction among MA in general [3, 36, 37, 39, 43], among MA working in methadone substitution clinics [34], among patient care teams [48], in comparison with general practitioners (GP) [50], as part of an intervention evaluation [41] or theoretically as a service-oriented profession [10]. The consistency of the results regarding overall job satisfaction, captured with instruments such as the Copenhagen Psychosocial Questionnaire (COPSOQ) and the Warr-Cook-Wall job satisfaction scale (WCW), was high: all studies capturing job satisfaction reported that MA are overall satisfied with their job. The specific working conditions assessed with job satisfaction scales are presented in the narrative summary below.

##### Job demands

We summarized under this category concepts related to workload, time pressure (e.g. “quantitative demands”, “stress”, and “workload", “amount of responsibility”), emotions (e.g. “demands for hiding emotions”, “emotional demands”), role ambiguity (e.g. “role clarity”, “role conflict”, “responsibilities within the practice team are clear”, “tasks are not sufficiently defined”, “competencies of MA are clearly defined”), and work-privacy conflict [3, 41, 43, 44, 46, 49, 50]. Applying mid-point cut-offs, we identified potential critical outcomes among MA regarding “stress” [43] and “workload” [62], and conflicting outcomes for “quantitative demands” [41, 50]. Studies capturing role ambiguity [36, 37, 41, 47] suggested that most MA did not perceive their role or responsibilities as ambiguous. Similarly, studies capturing emotional demands [41, 50] and work-privacy conflict [41, 44, 50] reported values that can be interpreted as uncritical.

One quantitative study examined, amongst other things, demands during the COVID-19 pandemic [40] and found that 50.0% of the participating MA reported an increase in workload due to the pandemic and 53.6% felt burdened by staff shortages. As for emotional demands, these presented the highest burden for MA during the pandemic, with e.g. 75.8% of MA reporting feeling burdened by a feeling of not being able to let patients down during the crisis [40].

##### Job control

We conceptualized “job control” as an umbrella term for concepts related to decision latitude (e.g. “degree of freedom at work”, “scope of action”, “freedom of working method”, “decision latitude”, “decision making”, “job control” [3, 37, 41, 43, 45, 46, 48–50, 62]), as well as for the constructs “predictability”, “work disruption”, and “job insecurity” [41, 46, 49, 50]. Most quantitative and mixed method studies reported, if available, an un-critical value (e.g. below the mid-point cut-off) among MA for the concepts “degree of freedom at work”, “freedom of working method”, “decision making”, and “scope of action” [3, 36, 37, 43, 45, 46, 48]. Two studies presented potentially critical values for “influence at work” and “decision latitude” [41, 50]. A further study on the needs of MA reported that 40.9% (*n* = 356) of the sample would like to have a greater scope of action and freedom of choice [35], whereas another one [62] presented a potentially critical outcome for job control according to our calculations. The latter study further stated that occupational stressors related to poor job control were particularly problematic (prevalence of ≥ 85.1%) [62].

##### Job resources

We conceptualized “job resources” as aspects related to social relations at work (e.g. “social support”, “social relations”, “sense of community”, “bullying”, “working atmosphere in the practice team”, “cooperation”), training (e.g. “offer for training”, “support by supervisor for training”), feedback and leadership (e.g. “feedback”, “leadership quality”, “supervisor”), and other job aspects that can have a positive impact on work (e.g. “workplace environment”, “meaning of work”, “amount of variety in job” and “possibilities for development”) [3, 35–37, 41, 43, 45–50].

In general, the concepts related to social relations received the most positive values among the psychosocial working conditions captured in the scrutinized studies [3, 36, 37, 41, 43, 45, 47, 50, 62]. According to our calculations, only one study presented a value below the critical cut-off for “social relations” [50].

The evidence regarding leadership can be considered mixed. While most studies reported favorably about leadership [37, 47, 49, 50], others [35, 37] presented, according to our calculations, a critical value for the categories “feedback” and “supervisor”. Another study reported that the majority of MA expressed the need for more understanding from their supervisor and the wish for the physicians (their supervisors) to have educational opportunities related to organizational leadership [35].

“Meaning of work”, i.e. the feeling that the work done is important [41, 50], was consistently rated as very high. “Amount of variety in job” [3, 36, 37, 43, 45, 48] was also consistently reported as positive, and another study [49] reported that 89.5% (*n* = 790) in their sample agreed that their work is varied.

One study examining further training reported that a third of MA found the available offer of training courses for MA to be sufficient or very good and the majority of MA expressed to receive support from their supervisor to attend continuing education courses [47]. Another study examined “benefits”, which covered continuous education and opportunities for career advancement [46], and reported a value which, according to our calculations, can be considered as potentially critical. Scharf et al. [35] reported that more than half of the MA participating in their study expressed the need to have more educational opportunities. The concept “possibilities for development” was captured by two studies [41, 50], both reporting satisfaction in this area.

##### Job rewards

Status-related rewards entail career opportunities, which we analyzed as resources, and job security, which we analyzed as part of job control. Financial rewards of MA have been captured using the concept “income” [3, 36, 37, 43, 45, 48]. These studies consistently reported low satisfaction levels for this category, with two studies [3, 43] reporting potentially critical values according to our calculations. Further, one study captured the relationship between additional qualifications and financial incentives, reporting that 35.8% (*n* = 98) of the participating MA saw a financial incentive from additional qualification, but only 24.8% (*n* = 68) MA expressed to have received a remuneration increase due to an additional qualification [36]. Another study reported that 87.0% (*n* = 759) of the participating MA expressed their wish to have a higher salary [35].

Socioemotional rewards include esteem or recognition [31]. Studies capturing the concept “recognition for work” consistently reported moderate satisfaction with this form of reward [3, 36, 37, 43, 45, 48].

The imbalance between effort and rewards, captured with the ERI-ratio, has been associated with stress at work [32]. The ERI-ratio was captured by two quantitative studies [42, 49], which consistently reported mean ERI-ratios above 1, indicating the presence of work stress among MA. Vu-Eickmann et al. [49] reported that the prevalence of work stress according to the ERI ratio was high among MA (73.77%). Fauser et al. [42] also reported overcommitment as a further dimension of the ERI-model, which following our calculations also falled under the critical cut-off category among MA.

#### Psychosocial working conditions of medical assistants in qualitative studies

A total of *n* = 10 studies examined psychosocial working conditions among MA using qualitative methods. Table 3 presents the concepts identified in each study, as well as a summary of their main findings. Most studies used an inductive approach (i.e., categories emanating from the data) for the analysis of the collected data, whereas two studies [55, 57] structured the categories deductively (i.e., following a pre-established theoretical framework), adding inductive concepts into a pre-established category system. We present a brief narrative summary of the key findings structured by occurring themes under the categories job demands, job control, job resources and job rewards.

##### Job demands

The workload of MA was consistently reported as high [56–58]. High workload was said to be promoted by a high patient volume combined with a shortage of staff [58]. MA further expressed to be subjected to stress due to a high workload, high time pressure, great amount of administrative work and confidentiality requirements [52, 56]. Increased documentation requirements were perceived to raise the time needed to complete administrative tasks and, as a consequence, MA felt to have less time for patients [58]. According to the researchers’ observations, a high work load and work intensity further prevented MA from taking appropriate breaks [55, 58]. It was reported that in one practice MA completed administrative tasks during lunch breaks [55] and, more generally, that MA tended to work extra hours which often remained uncompensated [58], having a negative impact on their private lives [54].

Qualitative studies that examined psychosocial working conditions of MA during the COVID-19 pandemic reported an increased workload [51, 53], with one study reporting MA being overwhelmed by the situation [53]. Studies examining the implementation of interventions reported barriers in MA taking up new tasks and/or responsibilities in the context of these interventions due to an already existing lack of time [52, 53].

In general, the behavior of some patients, colleagues and supervisors was considered to be stressful [52, 56, 58]. These behaviors were reported to include a lack of understanding or support by colleagues or superiors [52, 58], emotionally short-tempered employers, and negative changes in patient’s attitudes [58]. The combination of a personally difficult employer combined with a lack of social support in the team was considered to be particularly stressful [58].

MA with a migration background reported that they were often asked to assume the role of translators and cultural mediators by patients, superiors, or colleagues, adding to their already existing workload [38]. However, those MA did not perceive these additional demands as stressful [38].

##### Job control

Tsarouha et al. [57] reported that high work intensity lead to frequent interruptions and incomplete execution of tasks. Interruptions by colleagues and patients were consistently reported as frequent [55, 57, 58]. “Unplannable” events and carrying out different tasks and roles simultaneously were considered as stressful by MA in one study [55]. Interruptions were caused by phone calls, emergency patients, and by supervisors’ behavior [58]. Multi-tasking was reported as common, especially among MA working at the reception desk [57]. Work design was considered to influence the ability to decide when to take breaks, e.g. the visibility of MA across the reception desk hindered them from taking mini-breaks [55] and led to the feeling of being constantly under observation [57]. However, MA reported to have a certain degree of decision latitude in some sets of assigned tasks, which they considered to be a key resource [58].

##### Job resources

Appropriate decision latitude regarding the sequence of job-related activities was mentioned as an important resource by MA [57–59]. MA managed interruptions through prioritization, e.g. by asking patients to cue or by not answering phone calls while doing something else [57]. Flexibility of working hours and the supervisor’s support in these regards were reported as positive by MA [55].

Good relationships with co-workers and superiors were consistently reported as a key resource of MA [52, 55–59], they facilitated teamwork and planning [55, 58] and helped MA deal with difficult and stressful situations, overall contributing to happiness at the workplace [59]. A study reported that team meetings helped MA strengthen cooperation with colleagues [59].

Good relationships with patients, including positive interactions with them, were consistently reported as an overarching job-related resource for MA [52, 56, 58, 59].

Further resources included access to suitable work stations, equipment and software [57], the broad variety of activities that characterize the profession [58], and trust shown in the organization of the own set of assigned tasks [59].

##### Job rewards

One study reported that MA’s low salary constitutes the central aspect of the precarity of the MA profession, and that MA are dissatisfied with their income, especially when they compare themselves to other professions in the health care sector [54]. Obtaining further qualifications was considered to hardly have an impact on salary or career prospects [54]. Besides desire for a higher salary, it was reported that MA desire greater recognition by society, patients, and supervisors [58]. A study conducted during the COVID-19 pandemic reported that MA felt that their role as frontline workers did not receive adequate recognition [51]. Positive feedback and gratitude from patients contributed to MA’s satisfaction with their work [53, 59].

### Mental health among medical assistants

Among the reviewed studies, *n* = 8 captured mental health among MA. We present an overview of these studies, all of which used quantitative methods, in Table 4.

Burnout was captured by three studies using the Copenhagen Burnout Inventory (CBI) [41, 42] and the COPSOQ [50, 63]. Out of these, only one study [42] reported a potentially critical value for burnout according to our cut-off calculation.

Stress was captured by five studies with the concepts “cognitive stress symptoms” using the COPSOQ [41, 50, 63], “chronic stress” using the Trier Inventory for the assessment of Chronic Stress (TICS-SSCS) [39, 61], and “stress” using the Differential Stress Inventory (DSI) [60]. These studies reported consistently uncritical values when we applied a mid-point as cut-off. However, the prevalence of high strain among MA due to chronic stress was reported to be at 26.4% [61].

Two studies captured depressive symptoms using the short version of the Patient Health Questionnaire (PHQ-2) and generalized anxiety disorder symptoms using the Generalized Anxiety Disorder questionnaire (GAD-2) [40, 49]. The first study [49] reported no critical values according to our calculations for the sample average, but the prevalence of depressiveness among MA was 17.45% and for anxiety 20.14%. Similarly, a study conducted during the COVID-19 pandemic reported a prevalence of depressive symptoms of 29.9% and of generalized anxiety disorder symptoms of 42.6% among MA [40].

## Discussion

### Summary of evidence

This is to our knowledge the first scoping review on psychosocial working conditions and mental health among MA in Germany. Studies on this topic used to be sparse but have increased in numbers in the past years. The 30 reviewed studies used heterogeneous concepts and instruments. Compared to studies on psychosocial working conditions, studies on mental health among MA were rather scarce.

Quantitative and mixed methods studies consistently indicated that MA in Germany were satisfied with their job and considered their role and responsibilities to be clear. Studies examining work-privacy conflict, meaning of work, job insecurity, predictability, and amount of variety in job reported these consistently as unproblematic. We found a favorable tendency in reports on decision latitude as well as social relations at work. Findings were mixed regarding emotional demands, leadership, training and benefits, as well as on job demands. In quantitative and mixed methods studies, recognition for work was consistently reported as moderate, while satisfaction with income was consistently reported as low. Quantitative demands, stress and workload were partially reported as problematic, especially during the COVID-19 pandemic.

With the exception of the study by Hoffmann et al. [53], which reported that the examined intervention positively affected the job satisfaction among MA, “job satisfaction” was not directly referred to as a term in qualitative studies. Qualitative studies indicated more consistently that MA face job demands in terms of a high workload, high intensity, and time pressure, leading them to take few breaks or work overtime. The interaction with some patients and co-workers was also reported as demanding. Further, qualitative studies indicated that the everyday work of MA, especially during consultation hours, was characterized by interruptions, unplanned events, and the need for multi-tasking. Following qualitative studies, decision latitude regarding the sequence of job-related activities, support from colleagues and supervisors and positive relationships with patients constituted important job resources for MA.

Regarding mental health, most studies presented overall uncritical values, whereas the prevalence of mental health symptoms or high strain due to chronic stress, when measured, can be considered high when compared with the general female population in Germany before [64] and during the COVID-19 pandemic [65]. No prevalences were reported for burnout symptoms among MA.

### Quantitative versus qualitative studies

Our results on psychosocial working conditions among MA revealed some discrepancies between quantitative and mixed methods studies on the one hand, and qualitative studies on the other. The latter consistently emphasized that high workload, a lack of time to complete tasks, and interruptions lead MA to prioritize tasks, multitask, skip breaks, or work longer in order to fulfill their tasks. Quantitative and mixed methods studies, in contrast, were less consistent regarding negative psychosocial working conditions pertaining to job demands and job control, or even reported favorably about them. A possible explanation for the observed discrepancies may be that the COPSOQ [66] and WCW instruments [67], which were most commonly used to examine psychosocial working conditions among MA in Germany, do not capture some of the specific aspects identified in qualitative studies, that is, interruptions, multitasking, long working hours, breaks, and rewards (except for income). Further, according to qualitative studies, the relationship with patients was an important resource or stressor among MA, whereas quantitative or mixed method studies rarely examined this concept, often limiting social relations at work to those with colleagues and supervisors. Hence, while the job satisfaction scales commonly used in the research of MA in Germany may provide important initial insights on their working conditions, it seems that they are not sufficient to capture the whole spectrum of psychosocial working conditions among MA.

Similar discrepancies between negative working conditions reported in qualitative studies and high levels of job satisfaction reported in surveys have been previously observed among healthcare workers in Germany [68]. It has been suggested that the global approach of the instruments capturing job satisfaction causes a reporting bias towards positive results, as they tend to produce answers that are assumed to be socially desirable and fail to capture more nuanced perceptions of the working conditions [68]. This could also be a possible explanation in the case of MA.

### Low income versus job satisfaction

Although MA are generally satisfied with their job, low income represents the aspect they seemed to be the least satisfied with. This result was consistent throughout the various types of studies. To date, different collective agreements regulate MA’s salary and working hours but are not legally binding. As a consequence, MA’s salary often depends on their negotiation skills [54]. It has been suggested that because MA are already under enormous strain balancing their job and private life, they fail to participate in unions and organize themselves to claim better employment rights [54]. The overall job satisfaction and the higher satisfaction with other psychosocial working conditions such as social relations among MA could be interpreted as a possible reason for MA to accept their salary. It is also possible that MA not satisfied with their job have already left the profession and are not reflected in the results of this review, thus resulting in a possible selection bias. Such resignation or turnover have been described as possible explanations for the discrepancy between negatively perceived working conditions and high levels of job satisfaction among hospital staff in Germany [68]. A meta-analysis among nurses indicated that turnover was highly associated with supportive and communicative leadership, network centrality (i.e., being surrounded by meaningful social connections to peers), and organizational commitment, whereas salary had no significant effect [19]. To our knowledge, there is to date no study on actual turnover among MA.

### Working conditions during the COVID-19 pandemic

Outpatient practices had a key role during the COVID-19 pandemic in Germany, e.g. by contributing to a great extent to the national immunization campaign and by assuming other tasks, such as the information of patients regarding the pandemic, which were not adequately or completely covered by the responsible health authorities [69]. Three studies indicated a subjective increase in workload for MA in this setting due to the pandemic [40, 46, 51], whereas the only included study examining mental health among MA during this period [40] reported a very high prevalence of depressive symptoms (29.9%) and of generalized anxiety disorder symptoms (42.6%) among MA compared with the general female population in Germany during the pandemic [65]. A qualitative study from our working group published after our retrieval of sources and thus not included in this review highlighted the workload increase and unfavorable changes in the social interaction with colleagues and patients among MA during the COVID-19 pandemic [70]. These forms of negative impact on psychosocial working conditions and mental health have also been observed among other health care workers during the pandemic [71–75]. However, it has been suggested that physicians’ practices in Germany were less well prepared for the pandemic situation than hospitals [69] and that female health care workers were at higher risk of negative mental health outcomes during the pandemic [76]. This may place MA among the most vulnerable health care workers during the crisis and could explain why the prevalence of anxiety and depressive symptoms among MA during the pandemic was, unlike that of other health care professions in Germany, higher than among the general population [74, 77, 78]. However, the prevalence of depressiveness (17.45%) and anxiety (20.14%) among MA was already high before the pandemic compared with the general female population in the country, which corresponded to 11.3% for any mood disorder and 3.0% for generalized anxiety disorder [64]. During the pandemic, MA further resented that their profession did not receive the same attention and recognition from society and the government for their contribution during the pandemic as nurses did [51, 70], and expressed a decrease of job satisfaction [70].

### Recommendations for future research

Apart from highlighting gaps in the literature, our results emphasize the importance of combining qualitative research and quantitative studies into a single study (i.e., mixed methods). The selection of instruments or concepts to measure psychosocial working conditions should be adapted to the particularities of the profession under examination and can be derived from qualitative studies. For instance, as a patient-oriented profession, the relationship with patients should also be part of future quantitative assessments of psychosocial working conditions among the MA profession [9].

To better understand the discrepancy between the overall job satisfaction among MA and unfavorable psychosocial working conditions, such as a low salary, further studies should engage with MA turnover, as unsatisfied MA may have already left the profession and are potentially underrepresented in studies addressing job satisfaction. This could be done by surveying MA who are about to leave or already left the profession. Alternatively, a study following a cohort of newly trained MA over time could provide insights on the psychosocial working conditions and mental health statuses leading to turnover in the profession.

Our results suggest that the psychosocial working conditions and mental health among MA worsened during the COVID-19 pandemic. It is vital to keep in mind that these results reflect the situation during a crisis and may not apply to normal operations. Thus, further studies should examine if the deteriorated psychosocial working conditions and mental health of MA persist in the post-pandemic era.

### Limitations

Due to the heterogeneity of the concepts and instruments used in the reviewed studies, concepts are not very clear-cut, and the comparison of different studies was difficult. We used a self-defined critical cut-off based on the mid-point of the reported scales as a pragmatic way to address this limitation. Through this approach, we aimed to facilitate the identification of prominent stressors in relation to other stressors, not in absolute terms. However, the choice of the cut-off was arbitrary, and this self-developed approach could not be applied to studies not reporting mean values. Also, the approach is only statistical and does not consider that mid-points can carry different meanings. For example, whereas an “amount of variety of job” value on the WCW scale of job satisfaction above the mid-point cut-off indicates satisfaction with this aspect of the job, a “versatility” value from the KFZA-scale above the mid-point cut-off indicates agreement with the statement that the job is versatile, but not necessarily satisfaction with this job aspect. Further, as common in scoping reviews, we did not conduct a critical appraisal of the individual sources of evidence, nor did we extract details about the recruitment of study participants, e.g., response rates. This is because, unlike systematic reviews, scoping reviews are not intended to provide evidence to guide policies [21], but aim to provide an overview of available evidence regardless of its quality [79]. Further, the replicability of Google Scholar searches has been criticized [80]. We have addressed this limitation by uploading the results of our database retrieval on OSF (https://osf.io/2837w/?view_only=37d260e1618e4556891819fd6e054f61), as recommended [80].

## Conclusions

The psychosocial working conditions among MA in Germany have been captured with heterogeneous concepts and instruments, whereby the job satisfaction scales of the WCW and COPSOQ instruments were used most commonly. Mental health among this population was captured in only eight out of 30 studies which addressed anxiety symptoms, burnout, depressive symptoms, and stress using established instruments. The scrutinized studies indicate that MA in Germany were overall satisfied with their job, but reported mixed results regarding job demands, control, resources, and rewards. The studies reported consistently that MA were unsatisfied with their income and suggested that MA are subjected to stress due to an effort-reward imbalance. As for MA’s mental health, averages can be considered unproblematic, but prevalences appear to be high when compared with the German female population. Additionally, it will be interesting to see if the current staff shortages of MA will lead to positive changes of their working conditions in the future, such as higher salaries.

Our results indicate that quantitative studies have so far not adequately assessed the relationship with patients and the job control aspects related to interruptions and multitasking as part of the psychosocial working conditions of MA in Germany, both aspects that are characteristic of the MA profession and have been mentioned in qualitative studies. The discrepancy between overall job satisfaction, a low salary and stressful working conditions requires further examination, as job satisfaction appears to be too general a concept to capture the whole spectrum of the MA profession. Additionally, further research is required to assess if the psychosocial working conditions and mental health of MA in Germany remained changed after the COVID-19 pandemic, and to better understand the psychosocial working conditions and the mental health status among MA in Germany.

### Supplementary Information

**Additional file 1:** **Annex 1. **Protocol amendments and their justification.

**Additional file 2:** **Annex 2.** Full electronic search strategy used for PubMed (Medline).

## Data Availability

Not applicable.
