# Supplementary material for: Psychosocial working conditions and mental health among medical assistants in Germany: a scoping review
Source: BMC Public Health. 2024 Mar 6;24:716. doi: 10.1186/s12889-024-17798-2 (PMC10916249; doi:10.1186/s12889-024-17798-2)
Supplement: Supplementary file 1 — Additional file 1: Annex 1. Protocol amendments and their justification. [file 12889_2024_17798_MOESM1_ESM.docx]

**Annex 1** Protocol amendments and their justification.

| *Section* | *Protocol* | | *Amendment* | *Justification* |
| --- | --- | --- | --- | --- |
| *Exclusion criteria* | 4. Population medical assistants (MA) not included in the study | 4. Results do not apply to MA or to a population constituted by at least 50% MA | | To ensure that the included results refer to MA mostly. Several studies included MA in their population, but it was not clear if the results could be applied to them (e.g. when MA are part of a population “non-physician staff” including mainly nurses. |
| *Exclusion criteria* | 5. Concept psychosocial working conditions or mental health not the main focus of the study | | 5. Concept psychosocial working conditions or mental health not captured in the study | To include all identified information relevant to our concepts of interest. Several studies providing results on our concepts of interests did not focus mainly on them. |
| *Inclusion criteria* | 4. Population medical assistants included in the study | | 4. Results should apply to MA or to a population constituted of at least 50% MA | To ensure that the included results refer to MA mostly. |
| *Inclusion criteria* | 5. Concept psychosocial working conditions or mental health constitute the main focus of the study | | 5. Concept psychosocial working conditions or mental health is captured in the study | To include all identified information on our concepts of interest. |
| *Data extraction* | - Reviewer - Date - Author(s) of publication - Year of publication - Source origin/country origin - Aims/purpose - Defining characteristics of study participants and sample size (if applicable) - Type of study (e.g. qualitative, quantitative, mixed-methods) - Specific methodology - Concepts used to capture psychosocial working conditions or mental health (if applicable) - Instruments used to capture psychosocial working conditions or mental health (if applicable) - How outcomes were measured (if applicable) - Setting and context-related information - Authors’ conclusion - Key findings - Reviewer’s comments | | - Reviewer - Date - Title - Author(s) of publication - Year of publication - Journal/Publication type - Source origin/origin country (if other than Germany) - Aims/purpose - Sample size - Defining characteristics of study participants /Setting and context related information - Type of study (e.g. qualitative, quantitative, mixed-methods) - Concepts used to capture psychosocial working conditions or mental health (if applicable) - Instruments used to capture psychosocial working conditions or mental health (if applicable) - Outcomes of significance to our concepts of interest - Further findings of interest - Authors’ conclusion/Key findings (if applicable) - Reviewer’s comments | After piloting the table, we adjusted the data extraction table in order to obtain the best possible information to answer our research questions. |
|  |  | |  |  |
