# Supplementary material for: Psychosocial working conditions and mental health among medical assistants in Germany: a scoping review
Source: BMC Public Health. 2024 Mar 6;24:716. doi: 10.1186/s12889-024-17798-2 (PMC10916249; doi:10.1186/s12889-024-17798-2)
Supplement: Supplementary file 2 — Additional file 2: Annex 2. Full electronic search strategy used for PubMed (Medline). [file 12889_2024_17798_MOESM2_ESM.docx]

**Annex 2** Full electronic search strategy used for PubMed (Medline).

| PCC-Framework component | Full search string including limits used |
| --- | --- |
| Population | (((medical OR physician OR practice OR "health care") AND assistant*[tiab]) OR ((non-physician OR practice) AND staff[tiab]) OR "practice nurse*"[tiab] OR (medical AND (secretar* OR receptionist*[tiab])) OR "medical records administrator*"[tiab] OR "allied health occupation*"[mesh] OR ("health care assistant*" AND "family practice"[tiab]) OR "allied health personnel"[mesh]) |
|  | AND |
| Concepts | (stress*[tiab] OR (job AND demand* AND control[tiab]) OR ((organisational OR organizational) AND (justice[tiab] OR injustice[tiab])) OR “effort reward imbalance”[tiab] OR (“job-demand-control-support”[tiab] OR (job AND demand* AND control AND support[tiab])) OR (job-demand-resource*[tiab] OR job-demands-resource*[tiab] OR (job AND demand* AND resource*[tiab])) OR "job satisfaction”[tiab] OR (“mental health”[tiab]) OR depress*[tiab] OR resource*[tiab] OR stressor*[tiab] OR demand*[tiab] OR anxiety[tiab] OR (psychosocial AND working AND condition*[tiab]) OR “stress, psychological”[mesh] OR “burnout, professional"[mesh] OR “psychosocial strain*”[tiab]) |
|  | AND |
| Context | ((german*[tw]) OR (germany[mesh)) |
|  | Applied filter: Publication Year 2002-2022 |
